# Counting blood precursors

**DOI:** 10.7554/eLife.100373

**Published:** 2024-07-17

**Authors:** Sarah Duchamp de Chastaigne, Catherine M Sawai

**Affiliations:** 1 INSERM Unit 1312 Bordeaux Institute of Oncology, University of Bordeaux Bordeaux France

**Keywords:** hematopoiesis, hematopoietic stem, progenitor cells, HSPCs, stem cells, bone marrow, blood, Mouse

## Abstract

A new mathematical model can estimate the number of precursor cells that contribute to regenerating blood cells in mice.

**Related research article** Liu S, Adams SE, Zheng H, Ehnot J, Jung SK, Jeffrey G, Menna T, Purton LE, Lee H, Kurre P. 2024. Dynamic tracking of native polyclonal hematopoiesis in adult mice. *eLife* **13**:RP97504. doi: 10.7554/eLife.97504.

The body continuously regenerates new blood cells throughout an organism’s lifetime. This process, known as hematopoiesis, is maintained by rare hematopoietic stem and progenitor cells (HSPCs) that reside in the bone marrow and can develop into the various cell types found in blood. This process is essential for key physiologic processes like transporting oxygen to tissues, clotting to stop bleeding, and immune responses. A reduction in the number of these precursor cells – for instance, due to aging or illness – can lead to defects in hematopoiesis. However, it is poorly understood how many HSPCs are needed to maintain normal hematopoiesis in adult animals.

HSPCs are often studied by transplanting bone marrow cells from a donor animal to a recipient animal that has undergone chemotherapy or irradiation to kill its own hematopoietic cells. The ability of donor cells to regenerate the blood cells can then be measured. However, this does not necessarily reflect how HSPCs behave in their natural environment without interference (Broxmeyer et al., 2015). One way for researchers to overcome this is to genetically modify animals so that hematopoietic cells express a Confetti reporter gene, which can produce a protein that fluoresces either green, red, yellow or cyan (Ganuza et al., 2022; Ganuza et al., 2019; Ganuza et al., 2017; Henninger et al., 2017; Figure 1). The percentage of cells fluorescing a certain color in the blood of adults, and how much this number varies between different animals, is then used to estimate the number of precursors that contribute to hematopoiesis: high variability between animals is associated with low precursor numbers, whereas low variability is associated with high precursor numbers (Figure 1).

**Figure 1.**
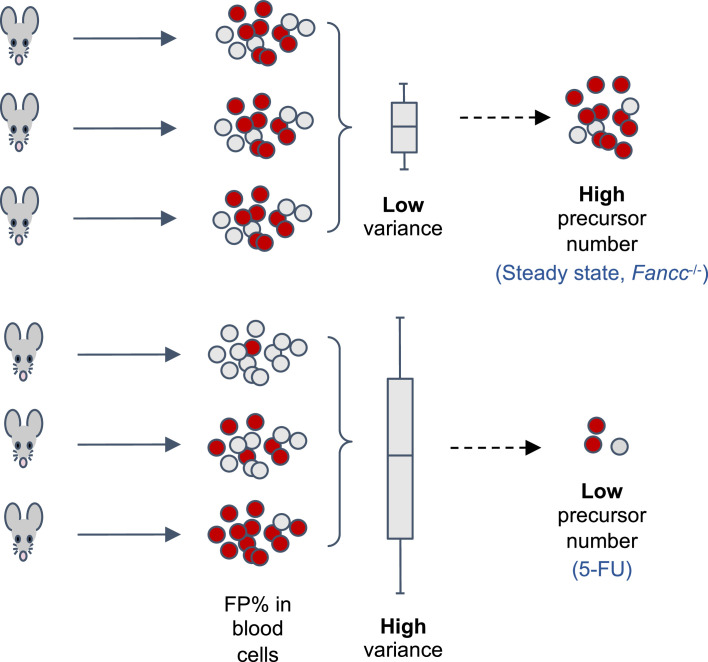
Using a fluorescent labelling system to estimate the number of precursor cells that contribute to hematopoiesis. The transgenic mouse model used by Liu et al. allows tamoxifen-inducible expression of one of four fluorescent proteins (FP) from a Confetti reporter in hematopoietic stem progenitor cells (HSPCs), which develop into the various cell types found in blood. The percentage of cells fluorescing a given color (in this case red) in the peripheral blood of the adult mice can be used to estimate the number of HSPCs contributing to hematopoiesis. How much this percentage varies between mice is inversely correlated with the number of HSPCs: low variation between animals correlates with a high number of precursor cells (top), whereas high variation is correlated with a low number of precursor cells (bottom). The percentage of fluorescent cells across different animals followed a binomial distribution, providing a mathematical basis to calculate the number of precursor cells. This revealed that hematopoiesis is associated with a high number of HSPCs in healthy adult mice (steady-state) and in mice genetically modified to have the bone marrow disease Fanconi Anemia (Fancc^-/-^). In addition, the ablation of bone marrow tissue with a drug called 5- fluorouracil (5-FU) reduced the number of precursor cells.

Experiments with the Confetti reporter system in mice revealed that hundreds of HSPCs in the embryo contribute to hematopoiesis in adults, and that expansion of these precursors is limited in the fetal liver (Ganuza et al., 2017; Ganuza et al., 2022). However, the previous mathematical model used to quantify the number of precursors was only tested for cell counts between 50 and 2,500 cells, which may not span the full range of HSPCs in mice (Cosgrove et al., 2021). Furthermore, the Confetti system has primarily been applied to hematopoiesis during development, and the complexity of the precursor pool in healthy adults remains poorly understood. Now, in eLife, Peter Kurre and colleagues – including Suying Liu as first author – report an updated mathematical formula for quantifying HSPCs that can measure a larger range of precursor numbers (Liu et al., 2024).

The team – who are based at the Children’s Hospital of Philadelphia, University of Pennsylvania and various institutes in Australia – found that variation in the percentage of cells expressing a given fluorescent protein could be mathematically modelled using a binomial distribution. They then developed a series of mathematical equations that use those data to estimate the number of precursor cells contributing to hematopoiesis.

To validate the mathematical model in vitro, Liu et al. cultured a known number of fluorescent and non-fluorescent human blood cells in individual wells. The population was allowed to proliferate and expand, and the percentage of cells that expressed the fluorescent protein was measured. As the number of initially cultured cells increased, the percentage of cells expressing the fluorescent protein became less varied between the wells. The calculated estimates were very close to the expected cell numbers for up to 100,000 cultured cells, indicating that the mathematical model developed by Liu et al. applies to a wide range of precursor numbers.

To confirm this model in vivo, Liu et al. used a transgenic mouse model that expresses the Confetti gene in all HSPCs following treatment with the drug tamoxifen (Göthert et al., 2005). A known number of bone marrow cells – which includes HSPCs – were then extracted from these donor mice and transplanted into recipient mice whose bone marrow cells had been destroyed through irradiation. Analysis showed that the inverse correlation between variation in the percentage of cells expressing a given fluorescent protein (in this case, red) and the number of precursors was also present in vivo.

The mice were transplanted with either a high or low number of bone marrow cells. Despite this difference, the ratio of donor and recipient cells and percentage of HSPCs in the bone marrow were similar across the two groups after a few months, indicating that low numbers of precursors can re-populate irradiated bone marrow as efficiently as high numbers. This highlights the general importance of being able to measure the number of active precursor cells, as simply measuring the percentage of donor cells in the blood does not directly reflect how many are actively contributing to hematopoiesis.

Next, Liu et al. used the mouse model expressing the Confetti system to study precursor numbers in different conditions. This showed that thousands of precursors contribute to steady-state hematopoiesis in adult animals, confirming that this pool of cells is derived from many different clones. The team also applied their mathematical formula to a mouse model with a reduced capacity to regenerate blood cells upon transplantation (Carreau et al., 1999; Whitney et al., 1996). This showed that the mice had a normal number of precursors, suggesting that their disease-associated characteristics are due to other factors, such as defects in proliferation (Figure 1).

The mathematical model developed by Liu et al. enables quantification of a wide range of precursor numbers and highlights the importance of cell numbers when studying hematopoiesis. While the number of HSPCs reflects the overall complexity of the precursor pool, this approach does not provide insight into the behavior of individual clones.

Like many lineage tracing systems, this model also depends on the use of tamoxifen, a compound that affects the proliferation and survival of HSPCs (Sánchez-Aguilera et al., 2014). Validating this model with an alternative fluorescent labelling system would clarify the potential impact of tamoxifen and reinforce the findings. Nevertheless, this mathematical model is compatible with any labelling system that follows the underlying premises of binomial distribution, facilitating its broad application in studying tissue regeneration at the steady state and during disease.
